# A donor-specific epigenetic classifier for acute graft-versus-host disease severity in hematopoietic stem cell transplantation

**DOI:** 10.1186/s13073-015-0246-z

**Published:** 2015-12-15

**Authors:** Dirk S. Paul, Allison Jones, Rob S. Sellar, Neema P. Mayor, Andrew Feber, Amy P. Webster, Neuza Afonso, Ruhena Sergeant, Richard M. Szydlo, Jane F. Apperley, Martin Widschwendter, Stephen Mackinnon, Steven G. E. Marsh, J. Alejandro Madrigal, Vardhman K. Rakyan, Karl S. Peggs, Stephan Beck

**Affiliations:** UCL Cancer Institute, University College London, London, UK; Department of Women’s Cancer, UCL Elizabeth Garrett Anderson Institute for Women’s Health, University College London, London, UK; Department of Haematology, University College London, University College London Hospital, London, UK; Anthony Nolan Research Institute, Royal Free Hospital, London, UK; Department of Haematology, University College London, Royal Free Hospital, London, UK; Clinical Immunology, Imperial NHS Trust Hammersmith Hospital, London, UK; Centre for Haematology, Faculty of Medicine, Imperial College London, Hammersmith Hospital, London, UK; Department of Clinical Haematology, Imperial College Healthcare NHS Trust, Hammersmith Hospital, London, UK; Blizard Institute, Barts and The London School of Medicine and Dentistry, Queen Mary University of London, London, UK

## Abstract

**Background:**

Allogeneic hematopoietic stem cell transplantation (HSCT) is a curative treatment for many hematological conditions. Acute graft-versus-host disease (aGVHD) is a prevalent immune-mediated complication following HSCT. Current diagnostic biomarkers that correlate with aGVHD severity, progression, and therapy response in graft recipients are insufficient. Here, we investigated whether epigenetic marks measured in peripheral blood of healthy graft donors stratify aGVHD severity in human leukocyte antigen (HLA)-matched sibling recipients prior to T cell-depleted HSCT.

**Methods:**

We measured DNA methylation levels genome-wide at single-nucleotide resolution in peripheral blood of 85 HSCT donors, matched to recipients with various transplant outcomes, with Illumina Infinium HumanMethylation450 BeadChips.

**Results:**

Using genome-wide DNA methylation profiling, we showed that epigenetic signatures underlying aGVHD severity in recipients correspond to immune pathways relevant to aGVHD etiology. We discovered 31 DNA methylation marks in donors that associated with aGVHD severity status in recipients, and demonstrated strong predictive performance of these markers in internal cross-validation experiments (AUC = 0.98, 95 % CI = 0.96–0.99). We replicated the top-ranked CpG classifier using an alternative, clinical DNA methylation assay (*P* = 0.039). In an independent cohort of 32 HSCT donors, we demonstrated the utility of the epigenetic classifier in the context of a T cell-replete conditioning regimen (*P* = 0.050).

**Conclusions:**

Our findings suggest that epigenetic typing of HSCT donors in a clinical setting may be used in conjunction with HLA genotyping to inform both donor selection and transplantation strategy, with the ultimate aim of improving patient outcome.

**Electronic supplementary material:**

The online version of this article (doi:10.1186/s13073-015-0246-z) contains supplementary material, which is available to authorized users.

## Background

Hematopoietic stem cell transplantation (HSCT) is a curative therapy for a wide range of hematological disorders and malignancies. Severe immune reactions, in particular graft-versus-host disease (GVHD), can decrease HSCT efficiency and survival in patients [1]. Immunosuppressive agents that counteract such events confer further complications, such as opportunistic infections and cancer recurrence [2].

Acute GVHD (aGVHD) has classically been described to develop within 100 days after HSCT, but can sometimes occur at later time points. In aGVHD, alloreactive donor T cells respond to antigens in the host tissues and damage recipient epithelial cells in skin, liver, and gastrointestinal tract [3]. T cell depletion of donor graft provides an efficient strategy of reducing the incidence of aGVHD, but can delay immune reconstitution and abrogate beneficial graft-versus-tumor effects [4]. Without T cell depletion, aGVHD affects 20–40 % of graft recipients when the donor and recipient are related, and 40–70 % when they are not related [5]. The incidence depends on a number of factors, including relatedness and degree of human leukocyte antigen (HLA) disparity, as well as differences in sex, age, and cytomegalovirus serostatus, between donor and recipient.

Promising novel therapeutic approaches to prevent or treat GVHD are being developed, including monoclonal antibodies targeting inflammatory cytokines and small-molecule inhibitors that alter immune cell trafficking (reviewed in [1]). In parallel, biomarkers that inform the risk of development and severity of GVHD are of substantial clinical importance. Several studies have measured plasma concentration levels of a number of different proteins, such as IL2RA and ST2, demonstrating correlation with responsiveness to treatment [6, 7]. Importantly, all biomarkers thus far characterized are applied in recipients after HSCT. Biomarkers that guide transplantation strategy have not yet been identified, but could provide a valuable approach to improving patient outcome following HSCT.

Epigenetic factors, such as DNA methylation and post-transcriptional modification of histones, play a critical role in regulating gene transcriptional programs that dictate immune cell fate and function [8]. Epigenetic mechanisms in circulating immune cells are sensitive to environmental factors and may contribute to disease development and progression, alongside genetic predisposition. For example, epigenetic mechanisms have been uncovered for distinct T cell differentiation pathways [9–11], and DNA methylation patterns have been associated with inflammatory and autoimmune disease susceptibility, including type 1 diabetes [12], systemic lupus erythematosus [13], and rheumatoid arthritis [14].

However, thus far little attention has been paid to the possible impact of epigenetic factors on HSCT outcomes. To this end, Rodriguez *et al.* examined DNA methylation differences in peripheral blood between donors and recipients (n = 47 pairs), both pre- and post-HSCT [15]. Global DNA methylation levels were estimated at CpG sites at repetitive DNA elements using a pyrosequencing-based assay. The results suggest that recipients maintain the donor’s global methylation levels after HSCT. DNA methylation levels were further measured at promoters of genes with functions relevant to immune responses in HSCT. In this analysis, the authors identified subtle DNA methylation changes at the *IFNG*, *FASLG*, and *IL10* gene promoters between recipients developing either no or mild, and severe aGVHD one month after HSCT.

Differential DNA methylation analyses between HSCT donors and recipients are impeded by several factors. First, recipients that are appropriate for HSCT suffer from a wide range of hematological malignancies. Epigenetic dysregulation in cancer etiology is well-described [16]; therefore, the meaningful comparison of DNA methylation patterns with regards to HSCT between healthy donors and patients is unattainable. Second, blood cells isolated from post-HSCT recipients may originate either from the remaining hematopoietic repertoire or the donor graft (that is, ‘mixed chimerism’), complicating the interpretation of the derived epigenetic signature.

In the present study, we investigated whether distinct epigenetic marks in peripheral blood of healthy graft donors delineate aGVHD severity in HLA-matched sibling recipients prior to HSCT. We measured DNA methylation levels genome-wide at 414,827 CpG sites at single-nucleotide resolution in peripheral blood of 85 HSCT donors, matched to recipients with various transplant outcomes. We defined a DNA methylation signature that stratifies graft donors with respect to aGVHD severity diagnosed in recipients, and replicated the signature with an alternative DNA methylation assay used in a routine clinical diagnostics environment. Here, we introduce the approach of epigenetic typing of HSCT donors to be used in conjunction with HLA genotyping to inform both donor selection and transplantation strategy.

## Methods

### Ethics

The research conformed to the Declaration of Helsinki and to local regulatory legislation. All HSCT donors and patients gave written informed consent according to local institutional guidance and JACIE (Joint Accreditation Committee of the International Society for Cellular Therapy and the European Group for Blood and Marrow Transplantation) standards for the analyses performed and publication of these data. The study was approved by the UCL Research Ethics Committee (Project ID 7759/001).

### Experimental design

The discovery cohort consisted of 85 HLA-identical sibling pairs who underwent reduced-intensity allogeneic HSCT between June 2000 and November 2012 at either the University College London Hospital or Royal Free Hospital (London, UK). Sibling pairs were 10/10 HLA allele-matched (that is, for *HLA-A*, *-B*, *-C*, *-DRB1*, and *-DQB1*). Donors provided peripheral blood stem cells mobilized by granulocyte-colony stimulating factor (G-CSF). All recipients received uniform conditioning with fludarabine, melphalan, and alemtuzumab [17]. Acute and chronic GVHD were assessed and graded according to published criteria [18]. Cyclosporine A was administered for GVHD prophylaxis. In the absence of GVHD, immunosuppression was decreased from three months after HSCT. The 85 graft donors were matched with recipients of various transplant outcomes: ‘severe’ aGVHD (grades III + IV; n = 9), ‘mild’ aGVHD (grades I + II; n = 37), and no aGVHD (n = 39). To obtain more equally powered sample groups, we enriched for severe transplant outcomes.

To assess the initial findings with regards to transplant conditioning regimen, we identified a validation cohort consisting of 32 HLA-identical sibling pairs undergoing T cell-replete HSCT between September 2000 and April 2012 at Hammersmith Hospital (London, UK). One of three regimens was used: (1) fludarabine alone; (2) fludarabine, rituximab, and cyclophosphamide; or (3) lomustine, cytarabine, cyclophosphamide, and etoposide. Patients received cyclosporine A and methotrexate as prophylaxis against GVHD. The 32 graft donors were matched with recipients of the following transplant outcomes: severe aGVHD (n = 9), mild aGVHD (n = 8), and no aGVHD (n = 15).

### DNA extraction

DNA was extracted from peripheral blood using a QIAamp DNA Blood BioRobot MDx Kit (QIAGEN) following the manufacturer’s instructions. The DNA concentration was assessed using a Qubit dsDNA BR Assay Kit (Invitrogen).

### Illumina Infinium HumanMethylation450 assay

Genomic DNA was bisulfite-converted using an EZ-96 DNA Methylation MagPrep Kit (Zymo Research) according to the manufacturer’s instructions. We applied 500 ng or 250 ng of genomic DNA to bisulfite treatment, and eluted purified, bisulfite-converted DNA in 20 μL or 11 μL of M-Elution Buffer (Zymo Research), respectively. DNA methylation levels were measured using Infinium HumanMethylation450 assays (Illumina) following the manufacturer’s protocol. In brief, 4 μL of bisulfite-converted DNA was isothermally amplified, enzymatically fragmented, and precipitated. Next, precipitated DNA was resuspended in hybridization buffer and dispensed onto Infinium HumanMethylation450 BeadChips (Illumina). To limit batch effects, samples were randomly distributed across slides and arrays. The hybridization was performed at 48 °C for 20 h using a Hybridization Oven (Illumina). After hybridization, BeadChips were washed and processed through a single-nucleotide extension followed by immunohistochemistry staining using a Freedom EVO Robot (Tecan). Finally, the BeadChips were imaged using an iScan Microarray Scanner (Illumina).

### Illumina Infinium HumanMethylation450 data preprocessing

The DNA methylation fraction at a specific CpG site was calculated as β = M/(M + U + 100), for which M and U denote methylated and unmethylated fluorescent signal intensities, respectively. The β-value statistic ranges from absent (β = 0) to complete DNA methylation (β = 1) at a particular CpG site. We normalized the 450K array data using Functional Normalization (FunNorm), a novel between-array normalization method, which is based on quantile normalization and uses control probes to act as surrogates for unwanted variation [19, 20]. In addition, the method entails background correction and dye-bias normalization using NOOB [21]. Next, we filtered: (1) probes with median detection *P* value ≥0.01 in one or more samples; (2) probes with bead count of less than three in at least 5 % of samples; (3) probes mapping to sex chromosomes; (4) non-CG probes; (5) probes mapping to ambiguous genomic locations [22]; and (6) probes harboring SNPs at the probed CG irrespective of allele frequency in the Asian, American, African, and European populations based on the 1000 Genomes Project (Release v3, 2011-05-21). All 450K array data preprocessing steps were carried out using the R package minfi [20]. Finally, we adjusted for batch effects (Sentrix ID) using an empirical Bayesian framework [23], as implemented in the ComBat function of the R package SVA [24]. The 450K array data generated as part of this study have been submitted to the European Genome-phenome Archive (https://www.ebi.ac.uk/ega/) with accession number EGAS00001001287.

### Estimation of differential leukocyte counts

We estimated differential leukocyte counts for each individual using an algorithm that is based on regression calibration [25], and implemented in the R package minfi [20]. In brief, for each sample the relative proportions of principal leukocyte cell types was inferred using DNA methylation signatures of an external validation set consisting of purified leukocytes, specifically CD3^+^CD4^+^ and CD3^+^CD8^+^ T lymphocytes, CD19^+^ B lymphocytes, CD56^+^ natural killer cells, CD14^+^ monocytes, and CD15^+^ granulocytes.

### Identification of differentially methylated regions (DMRs) and positions (DMPs)

We identified DMRs associated with aGVHD severity using Probe Lasso v6.1 [26]. We applied the following parameters: lassoStyle = max, lassoRadius = 2000, minSigProbesLasso = 2, minDmrSep = 1000, minDmrSize = 0, and adjPVal = 0.1. *P* values of DMRs were corrected for multiple testing with the false discovery rate (FDR) method. To identify DMPs, we fitted a linear regression model predicting DNA methylation state at each CpG site as a function of aGVHD severity (severe = 1 vs. no/mild aGVHD = 0), adjusted for sex, age at graft donation, and estimated differential cell counts (CD8T + CD4T + Bcell + NK + Mono + Gran). The DMP analysis was performed using the R package limma [27]. The approach uses an empirical Bayes method to moderate the standard errors of the estimated log-fold changes. *P* values of identified DMPs were corrected for multiple testing using the Bonferroni method.

### Annotation of DMRs using the Genomic Regions Enrichment of Annotations Tool (GREAT)

We analyzed the ontology of genes flanking the identified DMRs with GREAT v3.0.0 [28], using the standard parameters: association rule = basal + extension (constitutive 5 kb upstream, 1 kb downstream, up to 1 Mb extension); curated regulatory domains = included; background = whole genome.

### Assessment of epigenetic classifier performance with leave-one-out cross-validation (LOOCV)

To assess the performance of the epigenetic classifier, we used the preprocessed 450K array data set consisting of 85 donors. In each iteration of the LOOCV, one sample was left out and DMPs were identified using the remaining dataset (n = 85–1 donor samples). We used the same linear regression model, covariates, and significance thresholds for identifying DMPs as described above. Significant DMPs were ranked according to their *P* values. Then, a nearest shrunken centroid classifier was trained on the identified DMPs, as implemented in the R package pamr [29, 30]. The number of cross-validation folds was specified to the smallest class size, and a (random) balanced cross-validation was used (default parameters). The threshold for centroid shrinkage was set to one. The resulting centroid classifiers were used to predict aGVHD severity status on the omitted sample. Finally, classifier performance was evaluated using receiver operating characteristic (ROC) and area under the curve (AUC) measures, as implemented in the R package pROC [31].

### Measurement of relative DNA methylation levels using MethyLight

Genomic DNA was bisulfite-converted using an EZ DNA Methylation-Gold Kit (Zymo Research) according to the manufacturer’s instructions. PCR primers and probes for MethyLight analyses were designed specifically for bisulfite-converted DNA (5′ to 3′ plus strand) using ABI Primer Express v3. All oligonucleotides were synthesized by Metabion. Details with regards to PCR primers and probes used in this study are provided in Additional file 1. The reaction for the CpG of interest was assayed alongside a reference, the collagen 2A1 gene (*COL2A1*), to normalize for input DNA. Specificity of the reactions for methylated DNA was confirmed using M.SssI-treated human peripheral blood lymphocyte DNA (fully methylated positive control), whole-genome amplified DNA (unmethylated negative control), and a non-template control. The efficiencies of primers were assessed using a five-log serial dilution of M.SssI-treated human genomic standard. In addition, an agarose gel was run to ensure a single and appropriately sized PCR product. The fraction of fully methylated molecules at a specific locus was represented as percentage of methylated reference (PMR). First, all C_t_-values were interpolated from the standard curve based on a four-fold dilution of M.SssI-treated DNA. Then, we calculated PMR values by dividing the target CpG/reference C_t_-ratio of a sample by the CpG/reference C_t_-ratio of the M.SssI-treated DNA, multiplied by 100. All MethyLight reactions were performed on a 6FLX Real-Time PCR System (Life Technologies). DNA methylation thresholds with the maximal specificity and sensitivity were determined at the coordinates closest to the top-left part of the ROC curves (best.method = closest.topleft), as implemented in the R package pROC [31].

### Software for statistical analyses

All statistical analyses described in this study were performed using R v3.1.1 and Bioconductor v3.0.

## Results

### Characterization of distinct genome-wide DNA methylation signatures in HSCT donors

**Fig. 1:**
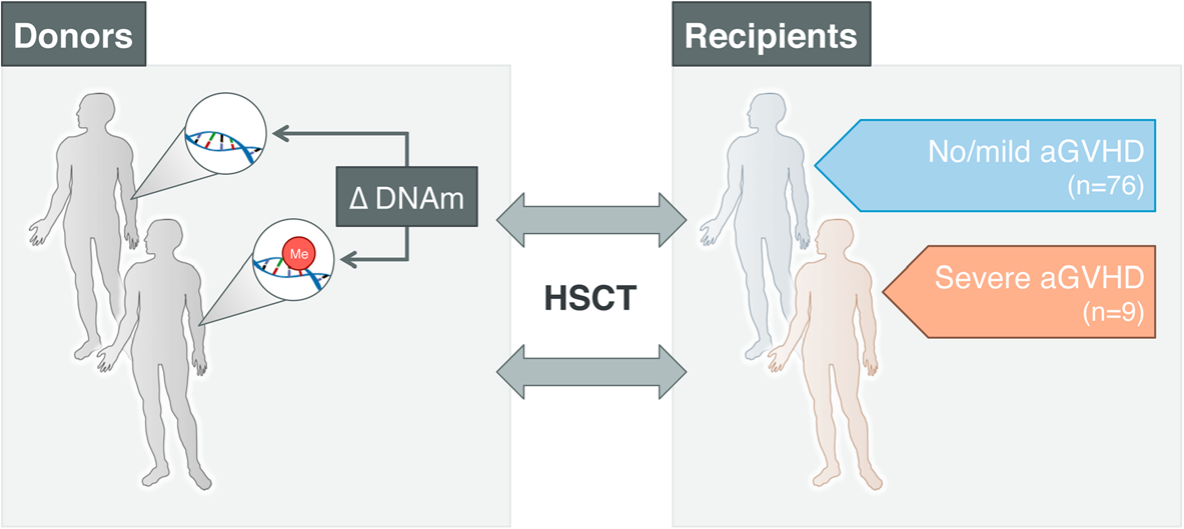
Overview of the study design. We aimed to identify specific epigenetic marks in peripheral blood of healthy graft donors that delineate aGVHD severity in HLA-matched sibling recipients prior to HSCT. At the discovery stage, we assessed genome-wide DNA methylation levels in peripheral blood of 85 HSCT donors, matched to recipients with various transplant outcomes, that is, ‘severe’ aGVHD (grades III + IV; n = 9) and ‘no/mild’ aGVHD (grades 0, I + II; n = 76). HSCT recipients received reduced-intensity (non-myeloablative) T cell-depleted conditioning using *in vivo* alemtuzumab. At the replication stage, we used a semi-quantitative DNA methylation assay, MethyLight, which can be easily used in a clinical setting. We validated the top-ranked differentially methylated positions associated with aGVHD severity status in donors in the context of both T cell-depleted and T cell-replete conditioning regimens for HSCT

We investigated a total of 85 HLA-identical HSCT donor-recipient sibling pairs. We focused on sibling pairs to minimize the contribution from genetic factors in our analyses. All patients undergoing HSCT received reduced-intensity (non-myeloablative) T cell-depleted conditioning using *in vivo* alemtuzumab. The T cell-depleted platform was chosen in the first instance in order to try to identify major drivers of aGVHD in the context of a platform with a relatively low incidence of GVHD. Sample selection was enriched for severe transplant outcomes to balance sample groups, that is, ‘severe’ aGVHD (grades III + IV; n = 9), ‘mild’ aGVHD (grades I + II; n = 37), and no aGVHD (grade 0; n = 39). Details about sample selection are described in the Methods section, and demographics of HSCT donors and recipients are provided in Table 1. An overview of the study design is illustrated in Fig. 1.

**Table 1:** Demographics of HSCT donors and recipients in the discovery and validation cohorts

	Discovery cohort		Validation cohort	
	(n = 85 pairs)		(n = 32 pairs)	
	Donors	Recipients	Donors	Recipients
Sex				
Female	41	27	14	7
Male	44	58	18	25
Age at transplant (years)				
Median	48	52	53	55
Range	14–72	21–66	22–71	23–64
CMV serostatus				
Positive	38	43	14	11
Negative	31	32	16	21
NA	16	10	2	0
Max. acute GVHD				
Absent	–	39	–	15
Grade I ('mild')	–	24	–	3
Grade II ('mild')	–	13	–	5
Grade III ('severe')	–	8	–	7
Grade IV ('severe')	–	1	–	2
Max. chronic GVHD				
Absent	–	54	–	2
Limited	–	21	–	10
Extensive	–	9	–	3
NA	–	1	–	17
Diagnosis				
Acute lymphoblastic leukemia	–	1	–	1
Acute myeloid leukemia	–	17	–	8
Chronic granulomatous disease	–	1	–	0
Chronic lymphocytic leukemia (CLL)	–	11	–	0
Chronic myeloid leukemia (CML)	–	0	–	3
Chronic myelomonocytic leukemia	–	1	–	0
Hodgkin lymphoma	–	9	–	4
Myelodysplasia (MDS)	–	2	–	0
MDS/CML/CLL	–	1	–	0
Myelofibrosis	–	0	–	2
Myeloproliferative syndromes/MDS	–	0	–	2
Myeloma	–	3	–	0
Non-Hodgkin lymphoma	–	37	–	8
Other	–	0	–	4
NA	–	2	–	0
	Sibling pairs		Sibling pairs	
Sex match (donor/recipient)				
Male/male	30		14	
Female/female	13		3	
Male/female	14		4	
Female/male	28		11	
CMV match (donor/recipient)				
Negative/negative	18		13	
Positive/positive	25		8	
Negative/positive	13		3	
Positive/negative	13		6	
NA	16		2	

Detailed information regarding criteria for study inclusion is provided in the Methods section. Note that the diagnosis ‘Non-Hodgkin lymphoma’ includes various disease subtypes.

*CMV* cytomegalovirus, *NA* data not available

We measured genome-wide DNA methylation levels in peripheral blood of HSCT donors using Illumina Infinium HumanMethylation450 BeadChips (‘450K arrays’). The two-color array allows the assessment of DNA methylation status at over 485,000 CpG sites at single-nucleotide resolution. The assay covers 99 % of RefSeq genes with an average of 17 CpG sites per gene region, and 96 % of CpG islands [32]. Array data preprocessing was performed using established analytical methods (Methods). Array probes were filtered with stringent quality criteria, leaving a total of 414,827 CpG sites for subsequent statistical analyses. A summary of the quality assessment of the 450K array data is shown in Additional file 2.

We performed multidimensional scaling (MDS) based on all measured CpG sites to assess the degree of similarity of individual HSCT donors. HSCT donors matched to healthy recipients and those matched to recipients diagnosed with mild aGVHD could not be discriminated using MDS (Additional file 2). Consequently, these two sample groups were combined for subsequent analyses. The analytical approach identified a DNA methylation signature that stratifies donors paired with recipients with severe aGVHD (Additional file 2).

To characterize the DNA methylation signatures underlying aGVHD severity, we identified DMRs between donors paired with no/mild aGVHD and severe aGVHD. DMRs have been shown to more likely locate near differentially expressed genes compared to differentially methylated single CpG sites [20]. DMRs were identified using the Probe Lasso algorithm [26], which applies a dynamic window based on probe annotation and density to record neighboring significant CpG sites and determine discrete DMR boundaries. A total of 453 DMRs at an FDR of less than 10 % were discovered. We annotated the genes flanking these DMRs using the Genomic Regions Enrichment of Annotations Tool (GREAT) [28], and observed enrichment in the ontology terms ‘MHC class II receptor activity’ (GO Molecular Function; *P* = 3.53 × 10^–5^, FDR-corrected binomial test), ‘MHC class II protein complex’ (GO Cellular Component; *P* = 4.46 × 10^–6^), ‘antigen processing and presentation’ (MSigDB Gene Sets Canonical Pathway; *P* = 2.08 × 10^–6^), ‘MHC classes I/II-like antigen recognition protein’ (InterPro; *P* = 1.97 × 10^–4^), among other relevant terms (Additional file 3). Taken together, these results indicate that healthy HSCT donors whose recipients develop severe aGVHD exhibit a specific DNA methylation signature, which correlates with known molecular processes relevant to GVHD pathobiology.

### Identification of differentially methylated positions associated with aGVHD severity

**Fig. 2:**
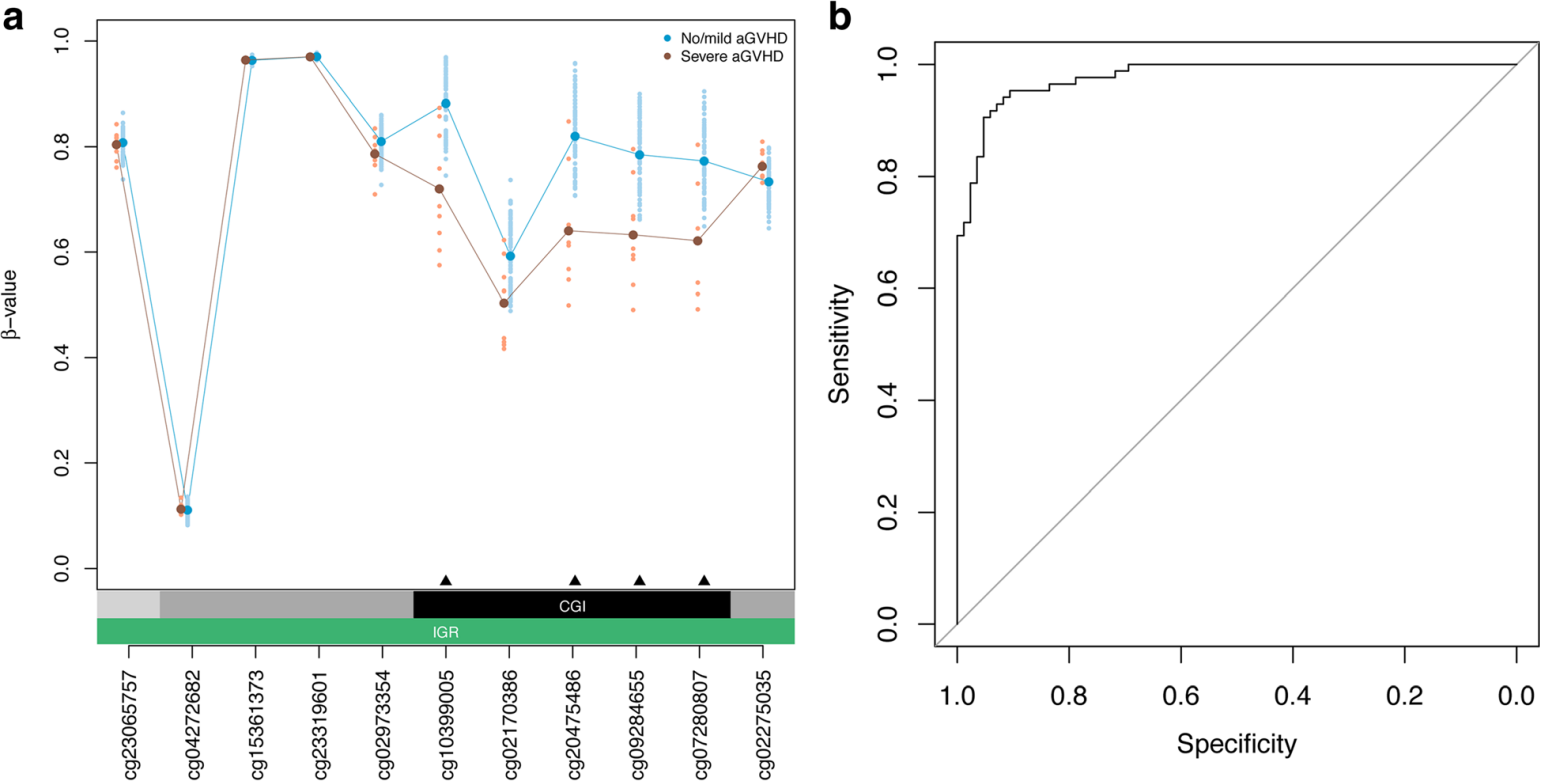
Identification of DMPs associated with aGVHD severity. **a** Genomic locus on chromosome 14q24.2 harboring four top-ranked DMPs associated with aGVHD severity. A DNA hypomethylation phenotype was observed in HSCT donors matched to recipients with severe aGVHD (red points) compared to donors paired with recipients with no/mild aGVHD (blue points). Lines represent the means of the measured DNA methylation levels (β-values) across HSCT donors. Statistically significant DMPs are indicated with a black triangle. Annotation of all significant DMPs is provided in Table 2. **b** ROC measures evaluating the epigenetic classifier performance. LOOCV was used to assess the classifier performance (Methods). Over 85 iterations of the LOOCV, the mean AUC was 0.98 (95 % confidence interval = 0.96–0.99), with a maximal specificity and sensitivity of 0.93 and 0.93, respectively

Next, we determined DMPs in HSCT donors associated with aGVHD severity in recipients that may be exploited as biomarkers for clinical diagnostics. We used a linear regression model predicting DNA methylation state at each CpG site as a function of aGVHD severity status, that is, severe vs. no/mild aGVHD diagnosed in matched graft recipients. In the regression model, we adjusted for sex, age at graft donation, and estimated relative proportions of major leukocyte cell types (Methods). We identified 31 DMPs that achieved a *P* value <0.05 after Bonferroni correction. To ascertain DMPs of potential biological significance, as well as permit validation using a semi-quantitative DNA methylation assay applicable for routine clinical testing, we only considered DMPs with a DNA methylation difference of at least ±5 % (Table 2). Strikingly, four top-ranked DMPs (that is, cg20475486, cg10399005, cg07280807, and cg09284655) form a DMR with consistent DNA hypomethylation in donors matched to recipients with severe aGVHD compared to donors paired with recipients with no/mild aGVHD (Fig. 2a). This locus was also identified as one of the top-ranking DMRs using the Probe Lasso algorithm (*P* = 4.55 × 10^–31^; rank = 2).

**Table 2:** Significant DMPs associated with aGVHD severity status

Rank	DMP	Adj. *P* value	Δβ-value	Chr.	Position	Gene	Feature	CpG context	LOOCV
1	**cg20475486**	1.38 × 10^−3^	−0.180	14	70,317,075	*–*	IGR	Island	85
2	**cg10399005**	1.62 × 10^−4^	−0.162	14	70,316,898	*–*	IGR	Island	85
3	cg16925210	1.60 × 10^−5^	0.130	2	216,946,718	*PECR*	TSS200	Island	85
4	cg00762468	7.61 × 10^−6^	0.066	16	103,568	*POLR3K*	1stExon	Island	85
5	cg26758857	1.07 × 10^−7^	−0.062	22	36,649,135	*APOL1*	5′UTR	–	85
6	cg21752471	8.92 × 10^−4^	−0.061	5	133,861,794	*PHF15*	TSS200	Island	85
7	cg21459486	5.14 × 10^−4^	−0.060	13	112,885,426	*–*	IGR	–	85
8	cg16495818	2.05 × 10^−5^	−0.055	11	73,306,367	*FAM168A*	5′UTR	Shelf	85
9	cg16276523	5.29 × 10^−4^	−0.094	1	161,049,579	*PVRL4*	Body	Island	84
10	cg19558933	5.11 × 10^−3^	−0.129	11	134,632,242	*–*	IGR	Island	82
11	cg00087067	9.63 × 10^−3^	0.071	5	5,265,509	*ADAMTS16*	Body	–	82
12	cg14099595	1.62 × 10^−2^	0.067	5	145,717,283	*POU4F3*	TSS1500	Shore	82
13	cg07464977	6.69 × 10^−3^	−0.062	5	2,097,136	*–*	IGR	–	82
14	cg11364888	8.50 × 10^−3^	−0.055	12	116,920,304	*–*	IGR	–	82
15	cg24599107	9.57 × 10^−3^	−0.073	1	161,049,443	*PVRL4*	Body	Island	81
16	**cg07280807**	8.94 × 10^−3^	−0.151	14	70,317,239	*–*	IGR	Island	80
17	cg14546466	2.49 × 10^−2^	−0.077	2	11,796,974	*–*	IGR	Island	80
18	cg03481039	8.60 × 10^−3^	−0.065	11	116,662,012	*APOA5*	Body	Shore	80
19	cg03896685	2.62 × 10^−2^	−0.062	7	65,512,561	*–*	IGR	Shelf	80
20	cg00245850	1.78 × 10^−2^	0.060	8	143,925,513	*GML*	Body	–	80
21	**cg09284655**	1.17 × 10^−2^	−0.152	14	70,317,228	*–*	IGR	Island	79
22	cg10287485	2.20 × 10^−2^	0.090	11	69,473,145	*–*	IGR	Shelf	79
23	cg19704238	1.55 × 10^−2^	0.058	10	85,900,022	*GHITM*	5′UTR	Shore	79
24	cg09282654	3.33 × 10^−2^	0.070	7	123,563,992	*SPAM1*	TSS1500	–	75
25	cg16701890	3.57 × 10^−2^	0.064	2	119,906,008	*–*	IGR	–	73
26	cg04664342	3.80 × 10^−2^	−0.107	17	4,685,905	*TM4SF5*	Body	Island	65
27	cg20307347	4.06 × 10^−2^	−0.119	2	231,734,563	*ITM2C*	Body	Shelf	58
28	cg19311448	4.17 × 10^−2^	−0.075	2	11,797,017	*–*	IGR	Island	45
29	cg09852744	4.55 × 10^−2^	0.058	6	158,732,256	*TULP4*	TSS1500	–	39
30	cg16398628	4.46 × 10^−2^	0.090	10	91,011,689	*LIPA*	TSS200	Island	32
31	cg20585841	4.57 × 10^−2^	−0.164	8	102,729,926	*NCALD*	Body	–	21

DMPs achieving a *P* value <0.05 after Bonferroni correction and a DNA methylation difference of at least ±5 % are reported. DMPs are ranked according to their recurrence in the LOOCV, for which a total of 85 iterations were performed, and their absolute DNA methylation difference. DNA methylation differences were calculated as follows: mean β-values of HSCT donors matched to recipients with severe aGVHD – mean β-values of HSCT donors matched to recipients with no/mild aGVHD. The four highlighted DMPs on chromosome 14q24.2 form a region with consistent DNA hypomethylation in graft donors matched to recipients with severe aGVHD compared to donors paired with recipients with no/mild aGVHD (Fig. 2a). Chromosomal positions are reported in genome build = hg19

To estimate the epigenetic classifier performance, we used leave-one-out cross-validation (LOOCV). In brief, one donor sample was left out in each iteration of the LOOCV, and DMPs were identified on the remaining sample cohort as described above. Then, a nearest shrunken centroid classifier was trained on the identified DMPs (Methods). The resulting classifiers were used to predict aGVHD severity status on the sample that was left out. Centroid classifier performance was evaluated by means of ROC curves and summarized by AUC values. Over the 85 iterations, the mean AUC was 0.98 (95 % confidence interval = 0.96–0.99; Fig. 2b). Importantly, all four DMPs contained within the DMR (Fig. 2a) were selected in over 90 % of iterations of the LOOCV classifier (Table 2). Our data indicate discovery of discrete DMPs that discriminate aGVHD severity status, and demonstrate strong predictive performance in internal cross-validation experiments.

### Replication of top-ranked differentially methylated positions using a clinical biomarker assay

Following the discovery of DMPs using 450K arrays, we aimed to replicate the top-ranked CpG sites using a semi-quantitative DNA methylation assay, MethyLight. This well-established assay uses PCR amplification of bisulfite-converted DNA in combination with fluorescently-labeled probes that hybridize specifically to a fully methylated DNA sequence [33]. The resulting data is presented as a percentage relative to an M.SssI-treated, fully methylated DNA reference sample (PMR). While the quantitative accuracy is lower compared to Illumina Infinium and next-generation DNA sequencing-based assays, MethyLight can be readily translated into a clinical setting at relatively low cost [34, 35].

**Fig. 3:**
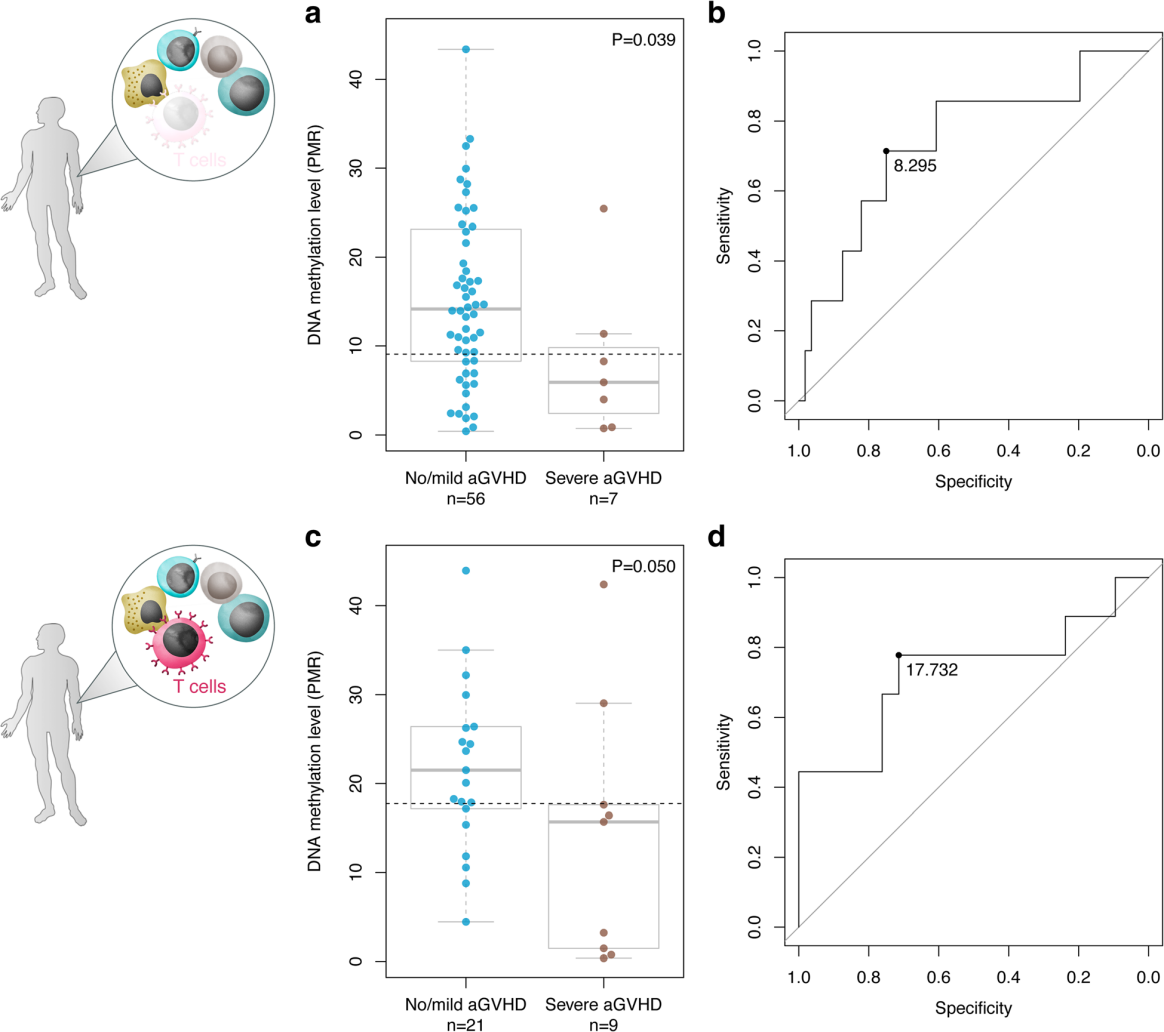
Validation of top-ranked DMP cg20475486 using a clinical biomarker assay. Replication of the top-ranked DMP associated with aGVHD severity, cg20475486, using a semi-quantitative DNA methylation assay. **a** Box-and-whisker plot of DNA methylation values in graft donors in T cell-depleted HSCT (initial discovery cohort). We replicated the DNA hypomethylation phenotype in HSCT donors matched to recipients with severe aGVHD compared to no/mild aGVHD (*P* = 0.039, Wilcoxon rank-sum test). **b** At a relative DNA methylation threshold of 8.295 (dotted line), the AUC was 0.74 with a maximal specificity and sensitivity of 0.75 and 0.71, respectively. **c** Box-and-whisker plot of DNA methylation values in graft donors in T cell-replete HSCT (that is, without the application of *in vivo* alemtuzumab). In an independent sample cohort, we confirmed the observed DNA methylation phenotype, suggesting the epigenetic classifier is also effective in the context of a T cell-replete conditioning regimen (*P* = 0.050). For two samples, C_t_-values could not be detected in the MethyLight experiments. **d** At a threshold of PMR = 17.73 (dotted line), the area under the ROC curve was 0.73 with a maximal specificity and sensitivity of 0.71 and 0.78, respectively

We focused our replication efforts on the highly discriminative DMPs located at the DMR on chromosome 14q24.2 (Table 2; Fig. 2a). We designed MethyLight reactions targeting three DMPs, cg20475486, cg10399005, and cg07280807. Through thorough assessment of the performance characteristics of the individual reactions (Methods), we identified cg20475486 with the highest PCR efficiency. Consequently, we measured relative DNA methylation levels at cg20475486 in 63 of the previous 85 HSCT donor samples, for which sufficient material was available. We replicated the observed DNA hypomethylation phenotype in donors paired with recipients diagnosed with severe aGVHD (*P* = 0.039, Wilcoxon rank-sum test; Fig. 3a). At a DNA methylation threshold with the maximal specificity and sensitivity, the AUC was 0.74 (Fig. 3b). Together, our results suggest technically robust identification of DMPs associated with aGVHD severity using both Infinium and MethyLight assays.

### Validation of epigenetic classifiers in donors in the context of T cell-replete HSCT

The discovery and replication of DMPs associated with aGVHD severity was carried out in donors matched to recipients that were subjected to T cell depletion as part of their transplant conditioning regimen. We next explored whether the identified epigenetic classifier could also be used in the context of T cell-replete HSCT (that is, without the application of *in vivo* alemtuzumab). We identified an independent sample cohort of 32 HLA-identical HSCT donor-recipient sibling pairs. As before, patients were selected based on transplant outcomes to obtain evenly numbered sample groups, that is, severe aGVHD (grades III + IV; n = 9), mild aGVHD (grades I + II; n = 8), and no aGVHD (grade 0; n = 15). Further details about sample selection and characteristics of HSCT donor-recipient sibling pairs are provided in the Methods section and Table 1, respectively.

In agreement with the data obtained in donors in the context of T cell-depleted HSCT, we confirmed the DNA hypomethylation phenotype at the top-ranked DMP cg20475486 (*P* = 0.050, Wilcoxon rank-sum test; Fig. 3c). The area under the ROC curve was 0.73 at the DNA methylation threshold with the maximal specificity and sensitivity (Fig. 3d). In summary, we validated the top-ranked DMP associated with aGVHD severity status in donors in relation to both T cell-depleted and T cell-replete conditioning regimens for HSCT. Our findings describe the first epigenetic classifier for the identification of donors with an intrinsically increased alloresponse prior to HSCT, identifying donor grafts more appropriate to undergo T cell depletion to reduce aGVHD incidence.

### Biological significance of DMR associated with aGVHD severity on chromosome 14q24.2

The DMR harboring the four top-ranked CpG classifiers (Fig. 2a) map to a CpG island at an intergenic region on chromosome 14q24.2. To investigate the potential functional role of this DMR, we annotated the genomic locus using available epigenomic reference datasets provided by the NIH Roadmap Epigenomics Project [36]. Specifically, we examined chromatin state maps of 22 primary hematopoietic cell types. Chromatin states are defined as spatially coherent and biologically meaningful combinations of distinct chromatin marks. These are systematically computed by exploiting the correlation of such marks, e.g., histone modifications, DNA methylation, and chromatin accessibility [37, 38]. This approach has recently been extended to include prediction (or ‘imputation’) of additional chromatin marks [39].

The annotation with both primary and imputed chromatin state maps revealed that the DMR is located at an active transcription start site or poised promoter in G-CSF-mobilized CD34^+^ hematopoietic stem cells, and a Polycomb-repressed region in CD3^+^ T cells of peripheral blood (Additional file 4). The closest annotated gene is *SMOC1* (SPARC related modular calcium binding 1), located 3.77 kb upstream of the top-ranked DMP cg20475486 (Table 2). *SMOC1* does not have a previously reported function in inflammatory or immune response pathways, and the evidence provided by the chromatin state maps suggests that *SMOC1* is unlikely to be the relevant target gene at this locus. Instead, the DMR may pinpoint a transcription start site of a novel, un-annotated gene or transcript that potentially plays a role in T cell lineage development.

## Discussion

GVHD is a condition in which both prevention and treatment are associated with significant costs and morbidities. In this study, we derived the first HSCT donor-specific DNA methylation signature that predicts the incidence of severe aGVHD in HLA-matched sibling recipients. Following a genome-wide survey in 85 HSCT donors using 450K arrays, we replicated the identified epigenetic signature associated with aGVHD severity status in 63 donors using MethyLight, a low-cost assay that is applicable for routine clinical diagnostics. Furthermore, we demonstrated the utility of the epigenetic classifier in the context of a T cell-replete conditioning regimen in an additional 32 HSCT donors.

We note that our study has limitations. Our DNA methylation analysis was carried out in peripheral blood, a substantially heterogeneous tissue. Cellular heterogeneity is a potential confounder in differential DNA methylation analyses [14, 40]. While we carefully assessed and controlled for differential leukocyte composition using statistical methods (Methods; Additional file 5), we cannot exclude the possibility that some of the identified DMPs are due to differential counts of cellular subpopulations that are not accounted for by the statistical inference. Indeed, previous studies have shown that most of the potential alloreactivity of a donor graft resides within the naïve T cell pool [41]. Therefore, differences in cellular composition of a subset of alloreactive T cells may even be anticipated. However, it should be noted that even if the associations are observed as a result of differential cell composition, this does not affect the validity of our finding as a valuable classifier.

The discriminatory performance of the presented epigenetic classifier, which consisted of only the CpG classifier cg20475486 at the replication stage, was reduced compared to the classifier panel consisting of multiple CpG sites at the discovery stage. We investigated whether variation in distinct HSCT donor groups (that is, donors matched to recipients with no complications, and those matched to mild aGVHD) caused the reduced performance, but could not substantiate this hypothesis (Additional file 6). Instead, the reason could be technical, because the 450K array platform measured DNA methylation levels at cg20475486 in single-nucleotide resolution, whereas MethyLight assessed the levels across eight linked CpGs (Additional file 1). Based on the combined graft donor pool across both T cell-depleted and T cell-replete HSCT (n = 93 donors; PMR = 8.295), the AUC was 0.69 with a maximal specificity and sensitivity of 0.81 and 0.56, respectively. The findings of our study will need validating in larger cohorts of HSCT donors that are matched to recipients with severe aGVHD. Also, we acknowledge that additional CpG classifiers are required to allow for effective routine clinical testing of graft donors prior to HSCT. Such additional CpG sites can be collated to constitute a more potent classifier panel, for example by drawing from the list of identified DMPs (Table 2). This strategy has previously been applied in an epigenetic biomarker panel for renal cell carcinoma [42] and active ovarian cancer [43] using 20 and even 2,714 distinct CpG classifiers, respectively. In addition, an independent discovery stage for HSCT donors whose recipients undergo T cell-replete conditioning may reveal a different set of DMPs.

The 450K array platform used for DNA methylation profiling holds a fixed, predesigned content covering less than 2 % of all annotated CpG sites. It is conceivable that CpG sites that are not captured by the array are more informative. Our study design also required two sample batches, which necessitated batch effect correction, potentially reducing the number of informative DMPs (Methods). Nonetheless, if combined with an alternative assay for replication of initial discoveries, 450K arrays are the current assay of choice for genome-wide surveys due to their quantitative, robust, and scalable assessment of DNA methylation levels.

We recognize that the presented epigenetic changes associated with aGVHD severity status may in fact merely mediate genetic risk factors for HSCT. We omitted array probes from statistical analyses that contained common genetic variants that likely influence DNA binding (Methods), but DNA methylation levels may be mediated by genetic variants in proximity, that is, represent DNA methylation quantitative trait loci (met-QTLs). Systematic met-QTL mapping efforts in HSCT donors with matched genotypic and epigenotypic datasets, combined with causal inference methods [14], are necessary to investigate this possibility further, but are beyond the scope of this study.

We provide evidence that DNA methylation signatures in graft donors associated with aGVHD severity in recipients correlate with well-characterized gene sets and molecular processes relevant to GVHD etiology, such as MHC class II restriction (Additional file 3). However, the functional importance of the specific DMR containing the four top-ranked CpG classifiers on chromosome 14q24.2 (Fig. 2a) is obscure and warrants additional experimental investigation. Annotation using chromatin state maps showed that the DMR maps to an active transcription start site in CD34^+^ hematopoietic stem cells, and a Polycomb-repressed regulatory element in CD3^+^ T cells (Additional file 4). Indeed, Polycomb proteins play a role in preventing the inappropriate hyperactivation of T cells in the setting of GVHD [44]. Future studies should delineate the DNA methylation signature in homogeneous T cell subsets and at various stages of T cell formation and development. International consortia, in particular BLUEPRINT [45], add further reference epigenomes of hematopoietic cell types, including many progenitor populations.

## Conclusions

Our results are the first to identify an epigenetic signature in healthy graft donors that can predict aGVHD in recipients. The findings suggest the possible use of epigenetic profiling in conjugation with genetic profiling to improve donor selection prior to HSCT and inform immunosuppressive transplant conditioning, with the paramount goal of improving patient outcomes. Looking ahead, we plan to further develop this first epigenetic classifier and its utility to also include unrelated HSCT donors, who constitute the majority of the allogeneic HSCT donor pool, and for which the incidence of aGVHD is most prevalent.

## Additional files

Additional file 1:
**PCR primers and probes used in MethyLight replication experiments.** A total of three MethyLight reactions were designed, which targeted top-ranked DMPs associated with aGVHD severity: cg10399005, cg20475486, and cg07280807. Of these reactions, cg20475486 achieved the highest PCR efficiency and was subsequently used in validation experiments (highlighted in gray). Start and end positions of primer and probes are noted in relation to the design start coordinates. Chromosomal positions are reported in genome build = hg19. (PDF 110 kb) Additional file 2:
**Quality assessment of the Illumina Infinium HumanMethylation450 assay.** The quality of the 450K array data was assessed after normalization, probe filtering, and batch correction (for details see Methods). (a) Density of DNA methylation β-values. (b) Multidimensional scaling (MDS) indicates the similarities and differences of samples by calculating the Euclidean distances between samples based on all CpG sites, and then projecting these distances into 2D coordinates. We found Dimension 4 to associate with aGVHD severity, accounting for 3 % of the total variance. HSCT donors matched to healthy recipients and those matched to recipients diagnosed with mild aGVHD could not be stratified using MDS. Therefore, these two sample groups were combined for subsequent analysis. (c) Singular value decomposition (SVD) determines the nature of the largest components of variation (Teschendorff AE et al. PLoS One. 2009;4:e8274). We assessed the first six principal components (PCs), and correlated these to phenotypic factors of donors (e.g., sex, age at transplant, and CMV serostatus), phenotypic factors of recipients (e.g., aGVHD status and severity), factors related to the experimental setup (e.g., Sentrix ID, sample plate, and sample well), as well as internal control parameters (e.g., bisulfite conversion efficiency). DNA methylation age (‘DNAm age’) was predicted based on the raw DNA methylation data using the DNA Methylation Age Calculator (https://dnamage.genetics.ucla.edu/), as described by Horvath (Horvath S. Genome Biol. 2013;14:R115). The phenotypic factor ‘transplant date’ denotes the year of the day of the graft transplant (day 0). We found PC4 and PC5 to most strongly correlate with aGVHD severity, achieving a significance level of *P* <0.01 and *P* <1 × 10^−5^, respectively. (PDF 507 kb) Additional file 3:
**Ontology annotation of genes flanking DMRs using GREAT.** We report the ontology of genes flanking the 453 identified DMRs. Specifically, we indicate all enriched terms in the binomial test over genomic regions at an FDR of less than 5 %. (PDF 107 kb) Additional file 4:
**Annotation of top-ranked DMPs using epigenomic reference datasets.** The genomic locus on chromosome 14q24.2 (position = 70,261,006–70,349,114; genome build = hg19) harboring the top-ranked DMR is shown using the WashU Epigenome Browser v40.0.0 (http://epigenomegateway.wustl.edu/browser/). The top-ranked DMR containing CpG classifiers of aGVHD severity (Table 2) is located at a CpG island (position = 70,316,847–70,317,240; indicated with an orange arrow). RefSeq and Gencode v17 genes, as well as CpG islands, are shown in the bottom panel of the figure. A total of 50 epigenomic reference tracks provided by the NIH Roadmap Epigenomics Project are displayed. Specifically, we show both the primary and imputed chromatin state maps in 22 primary hematopoietic cell types. The highlighted DMR overlaps with an active transcription start site (red) or poised promoter (pink) in G-CSF-mobilized CD34^+^ hematopoietic stem cells, and a Polycomb-repressed region in CD3^+^ T cells of peripheral blood. (PDF 960 kb) Additional file 5:
**Estimation of differential leukocyte counts.** For each sample, the composition of major leukocyte cell types was estimated using DNA methylation signatures of an external reference set consisting of purified leukocytes (Houseman EA et al. BMC Bioinformatics. 2012;13:86). The leukocyte composition was grouped for each measured cell type, and stratified for donors paired with healthy recipients (n = 39), recipients developing mild (n = 37) and severe aGVHD (n = 9). We did not observe significant differences (*P* <0.05) in cellular composition between the sample groups. For each cell type, the bar indicates the median of the composition estimate. Error bars indicate 95 % confidence intervals. *P* values were calculated using a Kruskal-Wallis rank-sum test. (PDF 235 kb) Additional file 6:
**Box-and-whisker plots of DNA methylation values in graft donors in the discovery cohort assessed using MethyLight and 450K arrays.** Only HSCT donors of the discovery cohort that were profiled on both assay platforms are shown. We identified a DNA hypomethylation phenotype at the top-ranked DMP cg20475486 in graft donors matched to recipients with severe aGVHD. HSCT donors matched to healthy recipients and those matched to recipients diagnosed with mild aGVHD could not be discriminated. (PDF 256 kb)

## Abbreviations

aGVHD: acute graft-versus-host disease
AUC: area under the curve
CGI: CpG island
DMP: differentially methylated position
DMR: differentially methylated region
DNAm: DNA methylation
FDR: false-discovery rate
G-CSF: granulocyte-colony stimulating factor
HLA: human leukocyte antigen
HSCT: hematopoietic stem cell transplantation
IGR: intergenic region
LOOCV: leave-one-out cross-validation
MDS: multidimensional scaling
MHC: major histocompatibility complex
PMR: percentage of methylated reference
ROC: receiver operating characteristic
TSS200/TSS1500: within 200/1,500 bp of a transcription start site
