# Supplementary material for: A donor-specific epigenetic classifier for acute graft-versus-host disease severity in hematopoietic stem cell transplantation
Source: Genome Med. 2015 Dec 15;7:128. doi: 10.1186/s13073-015-0246-z (PMC4681168; doi:10.1186/s13073-015-0246-z)
Supplement: Additional file 1: — PCR primers and probes used in MethyLight replication experiments. A total of three MethyLight reactions were designed, which targeted top-ranked DMPs associated with aGVHD severity: cg10399005, cg20475486, and cg07280807. Of these reactions, cg20475486 achieved the highest PCR efficiency and was subsequently used in validation experiments (highlighted in gray). Start and end positions of primer and probes are noted in relation to the design start coordinates. Chromosomal positions are reported in genome build = hg19. (PDF 110 kb) [file 13073_2015_246_MOESM1_ESM.pdf]

Additional file 1. PCR primers and probes used in MethyLight replication experiments. A total of three MethyLight reactions were designed, which targeted top-ranked DMPs associated with aGVHD severity, i.e. cg10399005, cg20475486, and cg07280807. Of these reactions, cg20475486 achieved the highest PCR efficiency and was subsequently used in validation experiments (highlighted in gray). Start and end positions of primer and probes are noted in relation to the design start coordinates. Chromosomal positions are reported in genome build = hg19.

DMP	Chr.	Position	Designed strand	Design Start Coord.	Reaction Start Coord.	Amplicon Length	Number of CGs	%Efficiency
cg10399005	14	70,316,898	Plus	70,316,698	70,316,858	76	9	80.3
cg20475486	14	70,317,075	Plus	70,316,698	70,317,047	116	8	**96.7**
cg07280807	14	70,317,239	Plus	70,316,698	70,317,199	134	3	84.0

DMP	Forward Primer Sequence (5'-3')	Length (bp)	Start Position	End Position	T_m	%GC
cg10399005	TTTTGTTGCGAGCGTGTGA	19	161	179	58.6	47
cg20475486	CGGAGAGCGTAGAGCGTATTTATT	24	350	373	59.0	46
cg07280807	GCGTAGTTAGGGATTATAGTGTAGTTGGT	29	502	530	58.6	41

DMP	Probe Primer Sequence (5'-3')	Length (bp)	Start Position	End Position	T_m	%GC
cg10399005	CGGCGGTAGTTGCGTGTTTCGTTTT	25	182	206	69.1	52
cg20475486	TTTCGGGTTTTGTTAATTGACGTTTGGTCG	30	375	404	69.6	40
cg07280807	TAGGTGGCGCGGAGGTTGTTGTG	23	533	555	68.8	61

DMP	Reverse Primer Sequence (5'-3')	Length (bp)	Start Position	End Position	T_m	%GC
cg10399005	CCGTATCCGCCTCATCGA	18	236	219	59.0	61
cg20475486	AACCGCCTAATTTCCGATAAAAA	23	464	442	59.0	35
cg07280807	CTATCTCCTCAAACAAAAACTAAAAACCT	29	635	607	58.2	31
