# Supplementary material for: A donor-specific epigenetic classifier for acute graft-versus-host disease severity in hematopoietic stem cell transplantation
Source: Genome Med. 2015 Dec 15;7:128. doi: 10.1186/s13073-015-0246-z (PMC4681168; doi:10.1186/s13073-015-0246-z)
Supplement: Additional file 2: — Quality assessment of the Illumina Infinium HumanMethylation450 assay. The quality of the 450K array data was assessed after normalization, probe filtering, and batch correction (for details see Methods). (a) Density of DNA methylation β-values. (b) Multidimensional scaling (MDS) indicates the similarities and differences of samples by calculating the Euclidean distances between samples based on all CpG sites, and then projecting these distances into 2D coordinates. We found Dimension 4 to associate with aGVHD severity, accounting for 3 % of the total variance. HSCT donors matched to healthy recipients and those matched to recipients diagnosed with mild aGVHD could not be stratified using MDS. Therefore, these two sample groups were combined for subsequent analysis. (c) Singular value decomposition (SVD) determines the nature of the largest components of variation (Teschendorff AE et al. PLoS One. 2009;4:e8274). We assessed the first six principal components (PCs), and correlated these to phenotypic factors of donors (e.g., sex, age at transplant, and CMV serostatus), phenotypic factors of recipients (e.g., aGVHD status and severity), factors related to the experimental setup (e.g., Sentrix ID, sample plate, and sample well), as well as internal control parameters (e.g., bisulfite conversion efficiency). DNA methylation age (‘DNAm age’) was predicted based on the raw DNA methylation data using the DNA Methylation Age Calculator (https://dnamage.genetics.ucla.edu/), as described by Horvath (Horvath S. Genome Biol. 2013;14:R115). The phenotypic factor ‘transplant date’ denotes the year of the day of the graft transplant (day 0). We found PC4 and PC5 to most strongly correlate with aGVHD severity, achieving a significance level of P <0.01 and P <1 × 10^−5^, respectively. (PDF 507 kb) [file 13073_2015_246_MOESM2_ESM.pdf]

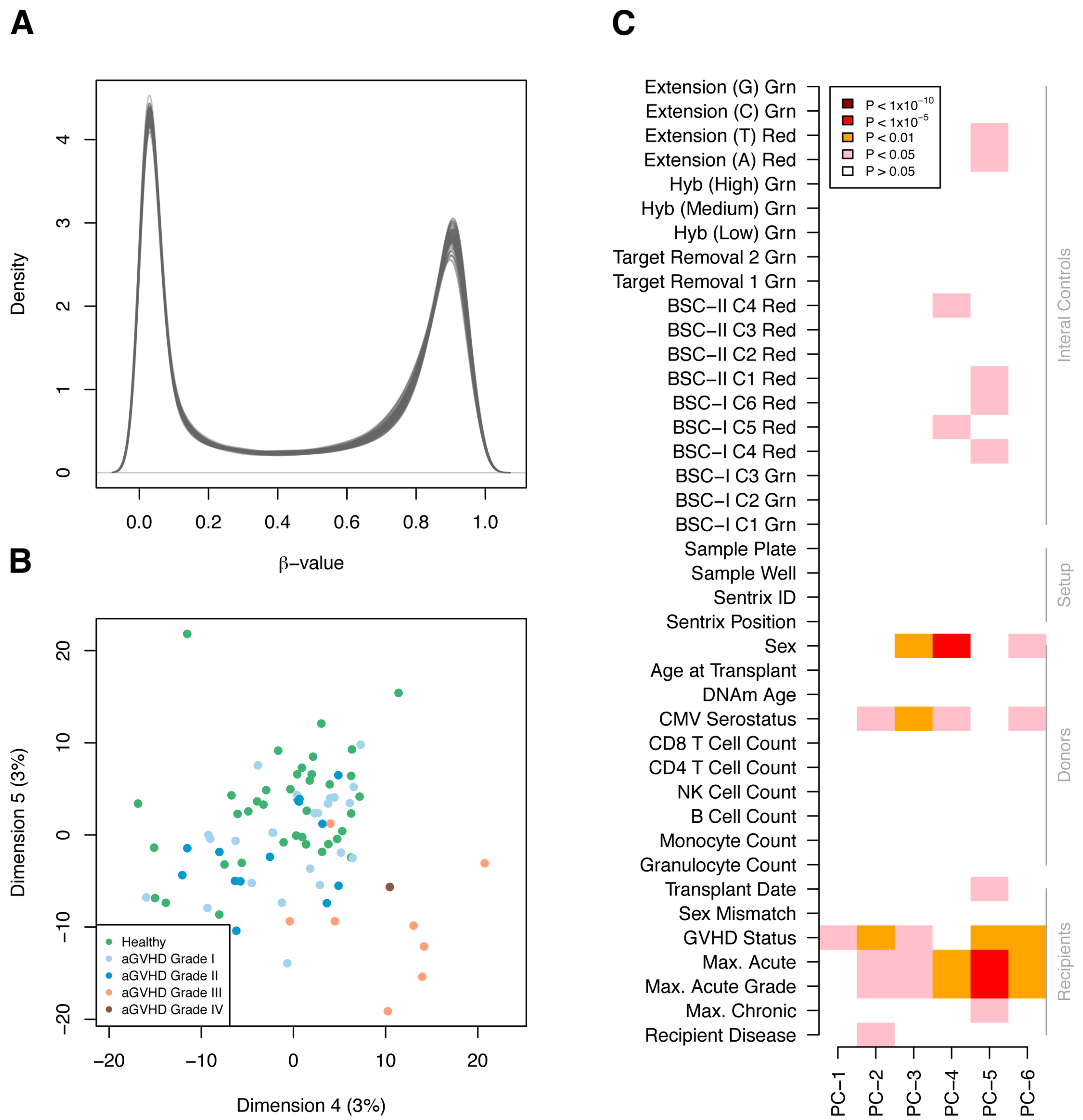

Additional file 2. Quality assessment of the Illumina Infinium HumanMethylation450 assay. The quality of the 450K array data was assessed after normalization, probe filtering, and batch correction (for details see Methods). (**A**) Density of DNA methylation β-values. (**B**) Multidimensional scaling (MDS) indicates the similarities and differences of samples by calculating the Euclidean distances between samples based on all CpG sites, and then projecting these distances into 2D coordinates. We found Dimension 4 to associate with aGVHD severity, accounting for 3% of the total variance. HSCT donors matched to healthy recipients and those matched to recipients diagnosed with mild aGVHD could not be stratified using MDS. Therefore, these two sample groups were combined for subsequent analysis. (**C**) Singular value decomposition (SVD) determines the nature of the largest components of variation (Teschendorff AE, et al. *PLoS ONE* 2009, **4:**e8274). We assessed the first six principal components (PCs),

and correlated these to phenotypic factors of donors (e.g. sex, age at transplant, and CMV serostatus), phenotypic factors of recipients (e.g. aGVHD status and severity), factors related to the experimental setup (e.g. Sentrix ID, sample plate, and sample well), as well as internal control parameters (e.g. bisulfite conversion efficiency). DNA methylation age ('DNAm age') was predicted based on the raw DNA methylation data using the DNA Methylation Age Calculator (https://dnamage.genetics.ucla.edu/), as described by Horvath (Horvath S. *Genome Biol.* 2013, **14**:R115). The phenotypic factor 'transplant date' denotes the year of the day of the graft transplant (i.e. day 0). We found PC4 and PC5 to most strongly correlate with aGVHD severity, achieving a significance level of $P < 0.01$ and $P < 1\times10^{-5}$, respectively.
