# Supplementary material for: A donor-specific epigenetic classifier for acute graft-versus-host disease severity in hematopoietic stem cell transplantation
Source: Genome Med. 2015 Dec 15;7:128. doi: 10.1186/s13073-015-0246-z (PMC4681168; doi:10.1186/s13073-015-0246-z)
Supplement: Additional file 3: — Ontology annotation of genes flanking DMRs using GREAT. We report the ontology of genes flanking the 453 identified DMRs. Specifically, we indicate all enriched terms in the binomial test over genomic regions at an FDR of less than 5 %. (PDF 107 kb) [file 13073_2015_246_MOESM3_ESM.pdf]

Additional file 3. Ontology annotation of genes flanking DMRs using GREAT. We report the ontology of genes flanking the 453 identified DMRs. Specifically, we indicate all enriched terms in the binomial test over genomic regions at an FDR of less than 5%.

Term Name	Rank	Raw *P*-value	Bonferroni *P*-value	FDR Q-value	Fold Enrichment	Expected / Observed Region Hits
**GO Molecular Function**						
MHC class II receptor activity	1	9.56×10^{-9}	3.53×10^{-5}	3.53×10^{-5}	42.41	0.141 / 6
**GO Cellular Component**						
MHC class II protein complex	1	3.52×10^{-9}	4.46×10^{-6}	4.46×10^{-6}	32.31	0.217 / 7
MHC protein complex	2	6.58×10^{-9}	8.32×10^{-6}	4.16×10^{-6}	21.33	0.375 / 8
Integral to lumenal side of ER membrane	3	7.45×10^{-9}	9.42×10^{-6}	3.14×10^{-6}	20.99	0.381 / 8
ER to Golgi transport vesicle membrane	4	4.15×10^{-5}	5.25×10^{-2}	1.31×10^{-2}	7.79	0.899 / 7
ER to Golgi transport vesicle	7	1.55×10^{-4}	1.96×10^{-1}	2.81×10^{-2}	6.29	1.114 / 7
Intrinsic to ER membrane	8	1.92×10^{-4}	2.43×10^{-1}	3.04×10^{-2}	3.56	3.372 / 12
**MSigDB Gene Sets Canonical Pathway**						
Antigen processing and presentation	1	1.57×10^{-9}	2.08×10^{-6}	2.08×10^{-6}	11.20	1.071 / 12
Type I diabetes mellitus	2	1.14×10^{-6}	1.50×10^{-3}	7.52×10^{-4}	7.65	1.307 / 10
Graft-versus-host disease	3	1.81×10^{-6}	2.38×10^{-3}	7.95×10^{-4}	10.11	0.792 / 8
Allograft rejection	4	5.61×10^{-6}	7.41×10^{-3}	1.85×10^{-3}	8.64	0.926 / 8
**InterPro**						
MHC classes I/II-like antigen recognition protein	2	4.17×10^{-8}	3.93×10^{-4}	1.97×10^{-4}	16.75	0.478 / 8
Immunoglobulin C1-set	3	6.70×10^{-8}	6.31×10^{-4}	2.10×10^{-4}	12.57	0.716 / 9
