# Supplementary material for: A donor-specific epigenetic classifier for acute graft-versus-host disease severity in hematopoietic stem cell transplantation
Source: Genome Med. 2015 Dec 15;7:128. doi: 10.1186/s13073-015-0246-z (PMC4681168; doi:10.1186/s13073-015-0246-z)
Supplement: Additional file 4: — Annotation of top-ranked DMPs using epigenomic reference datasets. The genomic locus on chromosome 14q24.2 (position = 70,261,006–70,349,114; genome build = hg19) harboring the top-ranked DMR is shown using the WashU Epigenome Browser v40.0.0 (http://epigenomegateway.wustl.edu/browser/). The top-ranked DMR containing CpG classifiers of aGVHD severity (Table 2) is located at a CpG island (position = 70,316,847–70,317,240; indicated with an orange arrow). RefSeq and Gencode v17 genes, as well as CpG islands, are shown in the bottom panel of the figure. A total of 50 epigenomic reference tracks provided by the NIH Roadmap Epigenomics Project are displayed. Specifically, we show both the primary and imputed chromatin state maps in 22 primary hematopoietic cell types. The highlighted DMR overlaps with an active transcription start site (red) or poised promoter (pink) in G-CSF-mobilized CD34+ hematopoietic stem cells, and a Polycomb-repressed region in CD3+ T cells of peripheral blood. (PDF 960 kb) [file 13073_2015_246_MOESM4_ESM.pdf]

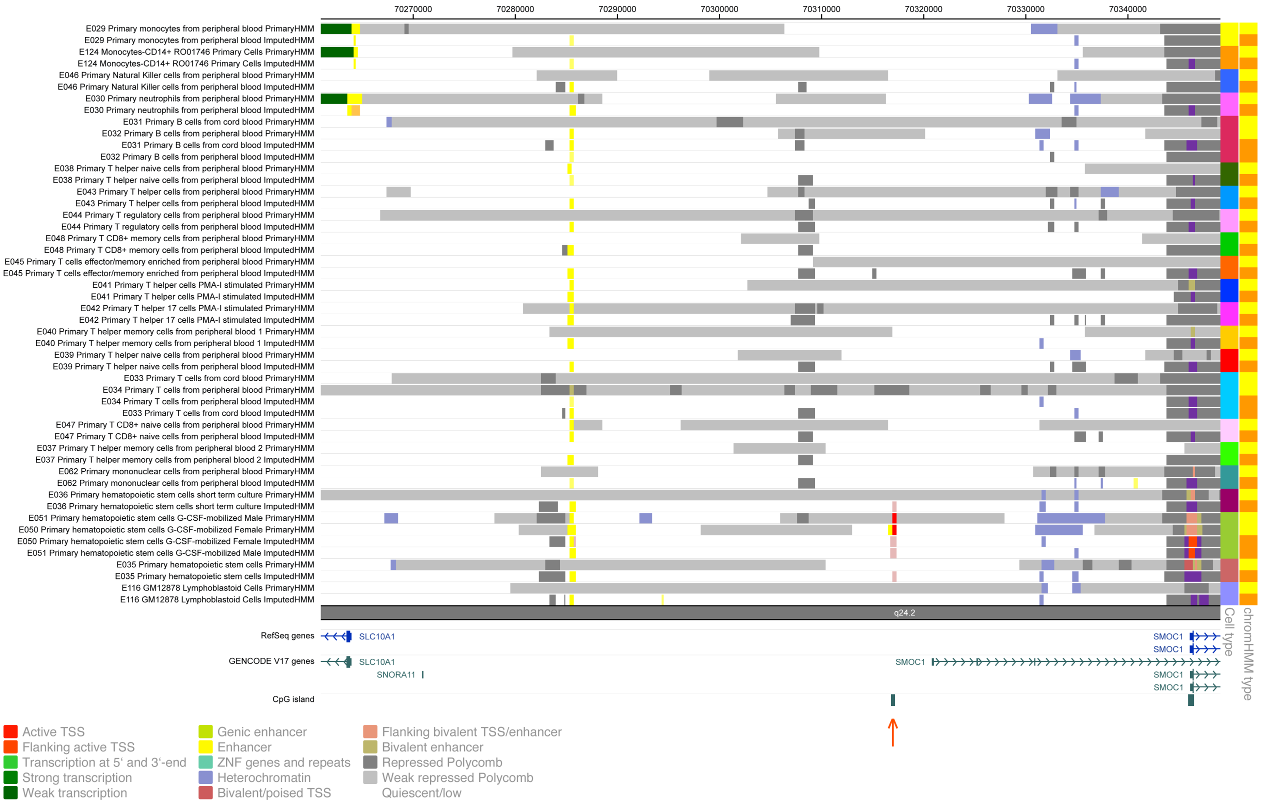

Additional file 4. Annotation of top-ranked DMPs using epigenomic reference data sets. The genomic locus on chromosome 14q24.2 (position = 70,261,006–70,349,114; genome build = hg19) harboring the top-ranked DMR is shown using the WashU Epigenome Browser v40.0.0 (http://epigenomegateway.wustl.edu/browser/). The top-ranked DMR containing CpG classifiers of aGVHD severity (Table 2) is located at a CpG island (position = 70,316,847–70,317,240; indicated with an orange arrow). RefSeq and Gencode v17 genes, as well as CpG islands, are shown in the bottom panel of the figure. A total of 50 epigenomic reference tracks provided by the NIH Roadmap Epigenomics Project are displayed. Specifically, we show both the primary and imputed chromatin state maps in 22 primary hematopoietic cell types. The highlighted DMR overlaps with an active transcription start site (red) or poised promoter (pink) in G-CSF-mobilized CD34$^+$ hematopoietic stem cells, and a Polycomb-repressed region in CD3$^+$ T cells of peripheral blood.
