# Supplementary material for: A donor-specific epigenetic classifier for acute graft-versus-host disease severity in hematopoietic stem cell transplantation
Source: Genome Med. 2015 Dec 15;7:128. doi: 10.1186/s13073-015-0246-z (PMC4681168; doi:10.1186/s13073-015-0246-z)
Supplement: Additional file 5: — Estimation of differential leukocyte counts. For each sample, the composition of major leukocyte cell types was estimated using DNA methylation signatures of an external reference set consisting of purified leukocytes (Houseman EA et al. BMC Bioinformatics. 2012;13:86). The leukocyte composition was grouped for each measured cell type, and stratified for donors paired with healthy recipients (n = 39), recipients developing mild (n = 37) and severe aGVHD (n = 9). We did not observe significant differences (P <0.05) in cellular composition between the sample groups. For each cell type, the bar indicates the median of the composition estimate. Error bars indicate 95 % confidence intervals. P values were calculated using a Kruskal-Wallis rank-sum test. (PDF 235 kb) [file 13073_2015_246_MOESM5_ESM.pdf]

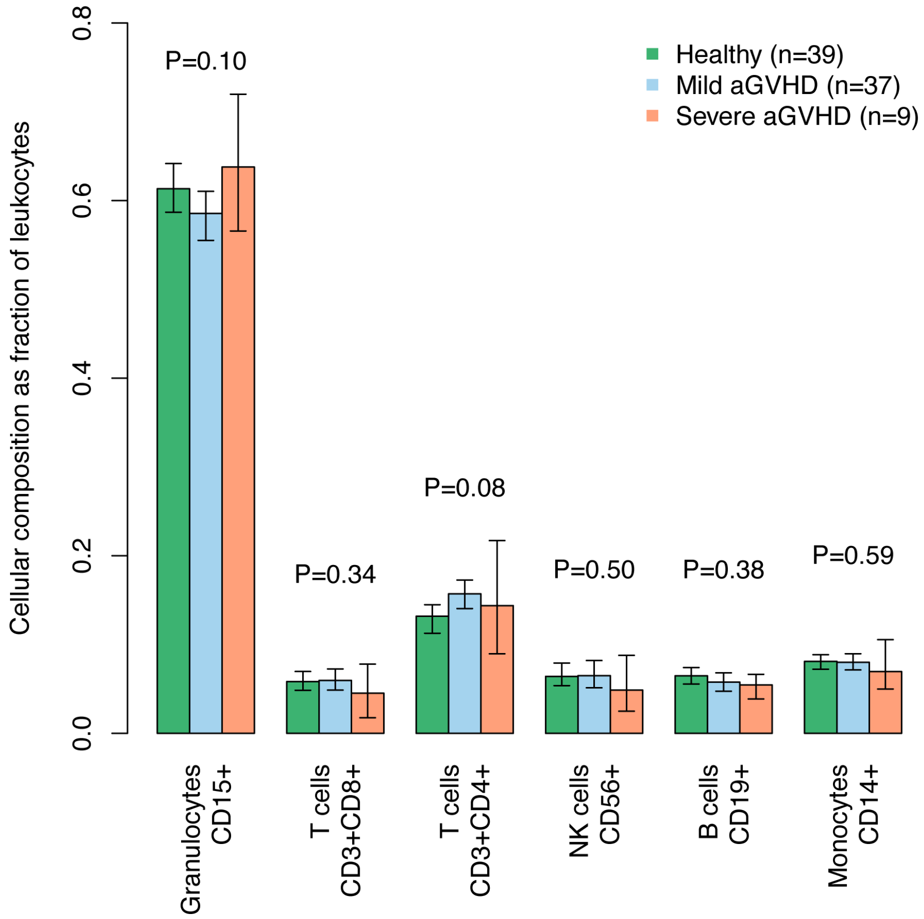

Additional file 5. Estimation of differential leukocyte counts. For each sample, the composition of major leukocyte cell types was estimated using DNA methylation signatures of an external reference set consisting of purified leukocytes (Houseman EA, et al. *BMC Bioinformatics* 2012, **13:**86). The leukocyte composition was grouped for each measured cell type, and stratified for donors paired with healthy recipients (n = 39), recipients developing mild (n = 37) and severe aGVHD (n = 9). We did not observe significant differences ($P < 0.05$) in cellular composition between the sample groups. For each cell type, the bar indicates the median of the composition estimate. Error bars indicate 95% confidence intervals. *P*-values were calculated using a Kruskal-Wallis rank-sum test.
