# Supplementary material for: A donor-specific epigenetic classifier for acute graft-versus-host disease severity in hematopoietic stem cell transplantation
Source: Genome Med. 2015 Dec 15;7:128. doi: 10.1186/s13073-015-0246-z (PMC4681168; doi:10.1186/s13073-015-0246-z)
Supplement: Additional file 6: — Box-and-whisker plots of DNA methylation values in graft donors in the discovery cohort assessed using MethyLight and 450K arrays. Only HSCT donors of the discovery cohort that were profiled on both assay platforms are shown. We identified a DNA hypomethylation phenotype at the top-ranked DMP cg20475486 in graft donors matched to recipients with severe aGVHD. HSCT donors matched to healthy recipients and those matched to recipients diagnosed with mild aGVHD could not be discriminated. (PDF 256 kb) [file 13073_2015_246_MOESM6_ESM.pdf]

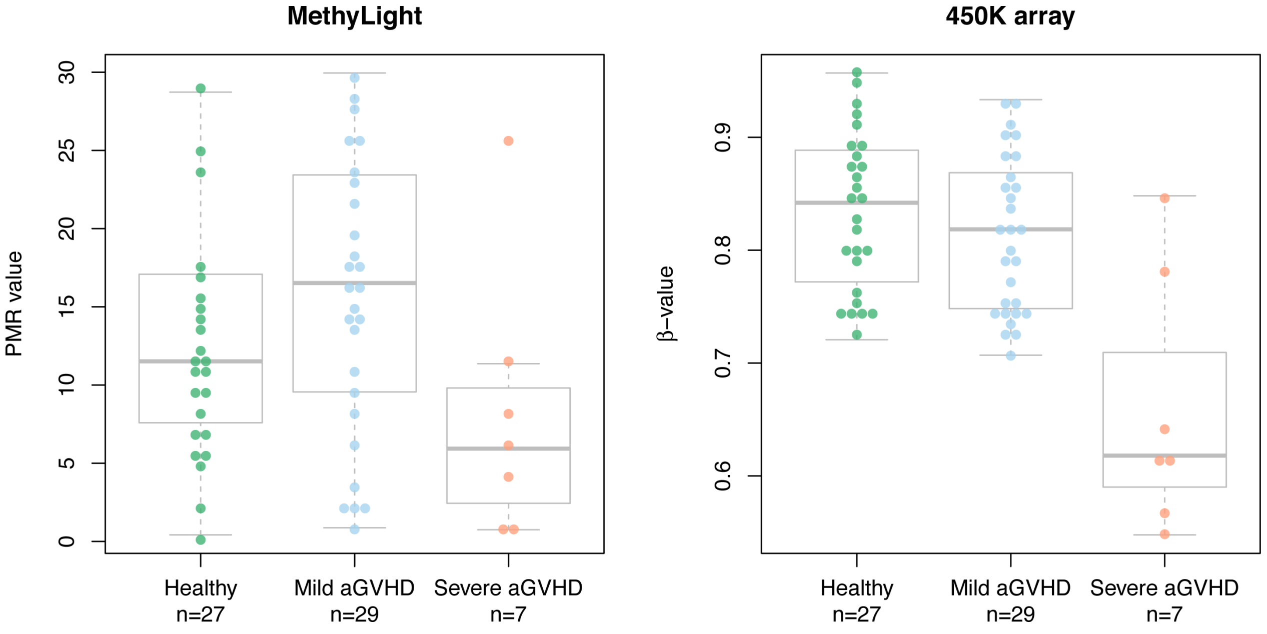

Additional file 6. Box-and-whisker plots of DNA methylation values in graft donors in the discovery cohort assessed using MethyLight and 450K arrays. Only HSCT donors of the discovery cohort that were profiled on both assay platforms are shown. We identified a DNA hypomethylation phenotype at the top-ranked DMP cg20475486 in graft donors matched to recipients with severe aGVHD. HSCT donors matched to healthy recipients and those matched to recipients diagnosed with mild aGVHD could not be discriminated.
